# NanoUPLC-MS^E^ proteomic data assessment of soybean seeds using the Uniprot database

**DOI:** 10.1186/1472-6750-12-82

**Published:** 2012-11-05

**Authors:** Andre M Murad, Elibio L Rech

**Affiliations:** 1 EMBRAPA Genetic Resources and Biotechnology, Synthetic Biology and Nanotechnology Group - Parque Estação Biológica, PqEB, Av. W5 Norte, 70770-917, Brasília, DF, Brazil

**Keywords:** Soybean, Seed proteomics, NanoUPLC-MS^E^, Uniprot database

## Abstract

**Background:**

Recombinant DNA technology has been extensively employed to generate a variety of products from genetically modified organisms (GMOs) over the last decade, and the development of technologies capable of analyzing these products is crucial to understanding gene expression patterns. Liquid chromatography coupled with mass spectrometry is a powerful tool for analyzing protein contents and possible expression modifications in GMOs. Specifically, the NanoUPLC-MS^E^ technique provides rapid protein analyses of complex mixtures with supported steps for high sample throughput, identification and quantization using low sample quantities with outstanding repeatability. Here, we present an assessment of the peptide and protein identification and quantification of soybean seed EMBRAPA BR16 cultivar contents using NanoUPLC-MS^E^ and provide a comparison to the theoretical tryptic digestion of soybean sequences from Uniprot database.

**Results:**

The NanoUPLC-MS^E^ peptide analysis resulted in 3,400 identified peptides, 58% of which were identified to have no miscleavages. The experiment revealed that 13% of the peptides underwent *in-source* fragmentation, and 82% of the peptides were identified with a mass measurement accuracy of less than 5 ppm. More than 75% of the identified proteins have at least 10 matched peptides, 88% of the identified proteins have greater than 30% of coverage, and 87% of the identified proteins occur in all four replicates. 78% of the identified proteins correspond to all glycinin and beta-conglycinin chains.

The theoretical Uniprot peptide database has 723,749 entries, and 548,336 peptides have molecular weights of greater than 500 Da. Seed proteins represent 0.86% of the protein database entries. At the peptide level, trypsin-digested seed proteins represent only 0.3% of the theoretical Uniprot peptide database. A total of 22% of all database peptides have a pI value of less than 5, and 25% of them have a pI value between 5 and 8. Based on the detection range of typical NanoUPLC-MS^E^ experiments, i.e., 500 to 5000 Da, 64 proteins will not be identified.

**Conclusions:**

NanoUPLC-MS^E^ experiments provide good protein coverage within a peptide error of 5 ppm and a wide MW detection range from 500 to 5000 Da. A second digestion enzyme should be used depending on the tissue or proteins to be analyzed. In the case of seed tissue, trypsin protein digestion results offer good databank coverage. The Uniprot database has many duplicate entries that may result in false protein homolog associations when using NanoUPLC-MS^E^ analysis. The proteomic profile of the EMBRAPA BR-16 seed lacks certain described proteins relative to the profiles of transgenic soybeans reported in other works.

## Background

Soybean *Glycine max* (L) Merrill] is one of the most important leguminous crops in the world with a vital importance to the economies of many countries. Brazil is responsible for 27% of the world soybean production and is second only to the U.S., which produces 35% [1]. Soybean seed products are used in a variety of industrial goods derived from oil (58%) and protein (68%) and are used to feed both humans and animals [1].

In the last decade, efforts have been undertaken to improve soybean crop yields. To this end, genetic engineering has been extensively used to develop soybean plants with abiotic and biotic resistance or tolerance [2]. However, both the quantity of grain produced and the nutritional content of the grain are critical; therefore, the production of highly nutritional seeds of many important crops is currently a focus of research [3-5]. Furthermore, the soybean is also a viable platform for the production of recombinant pharmaceutical molecules, such as human growth hormone [6] and coagulation factor IX [7], for several reasons: the soybean can undergo long-term storage at ambient temperatures [8,9], can provide an appropriate biochemical environment for protein stability through the creation of specialized storage compartments [9,10], is not contaminated by human or animal pathogens [8,11], its desiccation characteristics prevent it from undergoing non-enzymatic hydrolysis or protease degradation [11], and it does not carry harmful substances that are present in certain plant leaves, which is important for downstream processing [11,12].

To enhance protein content analysis efforts, the use of technologies that permit the analysis of protein expression patterns has become a necessity in evaluating the genetic modification of these plants [5,13-15]. The seed, leaf and root proteins of a variety of cultivars have been well documented [15,16]. Two-dimensional gel electrophoresis (2DE) is the most commonly used technique in proteomic analysis, and many types of proteomic studies based on 2DE have been reported [17]; however, 2DE is an extremely time-consuming technique. High throughput protein identification via 2DE requires the use of replicate gels as well as gel excision and digestion procedures [18]; these steps can be complicated and slow. Database comparisons are typically performed using peptide mass fingerprinting [19,20], and quantization is performed by gel image intensity evaluation or by protein tagging [21,22]. All of these stages of 2DE are time-consuming and can produce inconsistent results.

The coupling of liquid chromatography with mass spectrometry, as in NanoUPLC-MS^E^ procedures, provides more robust throughput sample analysis capabilities than other techniques. Complex samples may be prepared in single vials, and all processes associated with chromatography, MS and MS/MS acquisition and database searching can be performed in a few steps [23]. These experiments have led to significant innovations, such as the ability to obtain linear sequence structural information at the femtomole level [24], small surface areas and minimal dead volumes, which minimize analyte losses due to surface adsorption, as well as low flow rates, which minimize the required analyte dilution [25]. Low-abundance analytes can be separated with a high recovery rate when they are associated with a high dynamic range and a high-quality MS detection system [26]. In this present study, we used MS^E^, which is a data-independent acquisition method that uses low and high collision energies without precursor selection, unlike other methods such as data-dependent acquisition (DDA) [27]. Ion detection, clustering and the normalization of data-independent, alternate scanning LC-MS^E^ data have been explained in detail elsewhere [27,28].

Here, we present a statistical assessment of soybean seeds using NanoUPLC-MS^E^ proteomic experiments and provide a comparison with the theoretical tryptic digestion of sequences from the Uniprot [29,30] soybean database.

## Results and discussion

### NanoUPLC-MS^E^ proteomics

**Figure 1:**
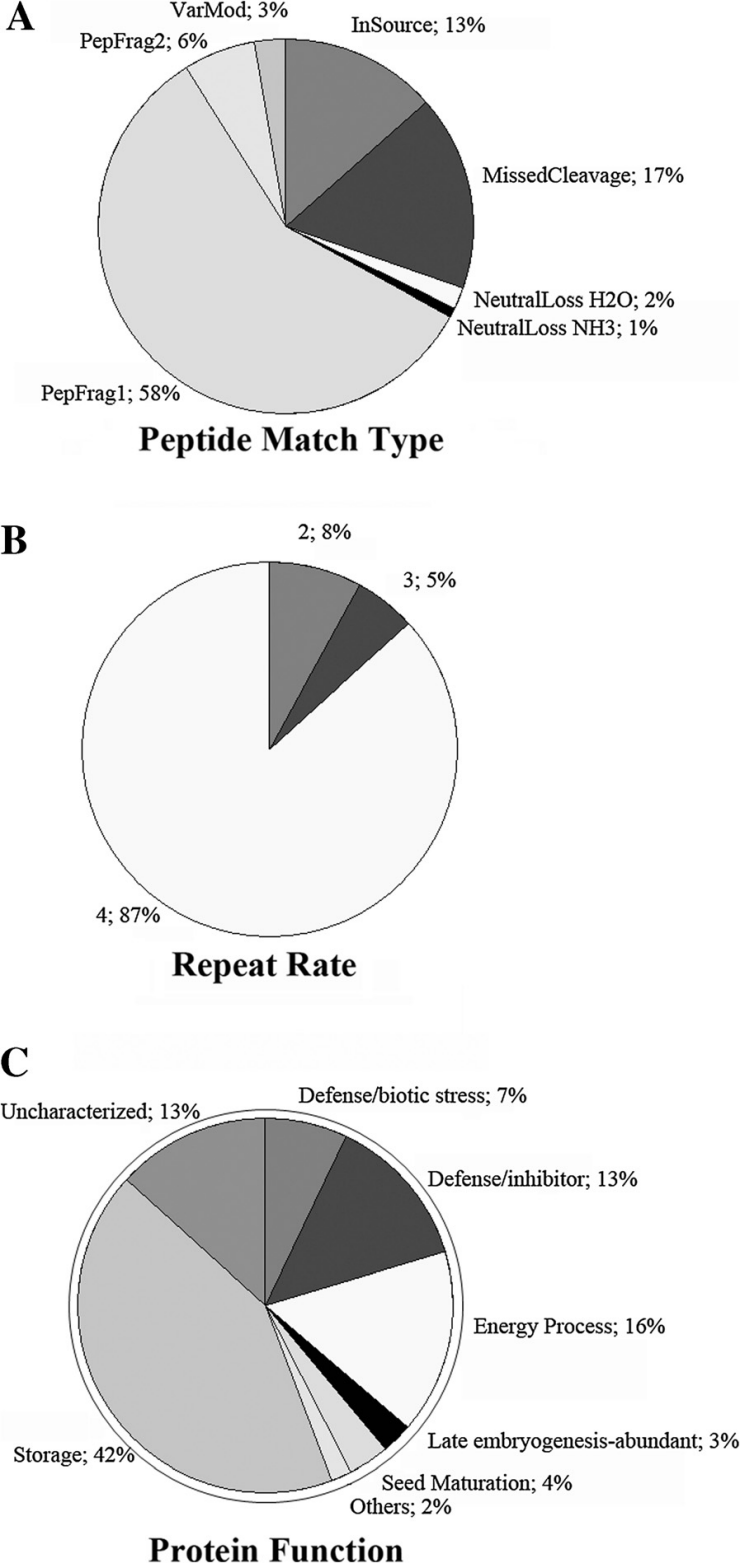
**Peptide detection type, repetition rate, and protein function chart.A**) On peptide match type, PepFrag1 and Pepfrag2 correspond to the peptide matches when compared to database by PLGS, VarMod corresponds to variable modifications, InSource corresponds to fragmentation that occurred on ionization source, MissedCleavage indicates the missed cleavage performed by trypsin and Neutral loss H2O and NH3 correspond to water and ammonia precursor losses; **B**) Repeat rate indicates the number of times that an identified protein apears on the replicas; **C**) Protein function of the identified proteins clustered in storage, defense, energy processing, embryogenesis, seed maturation or other functions.

**Figure 2:**
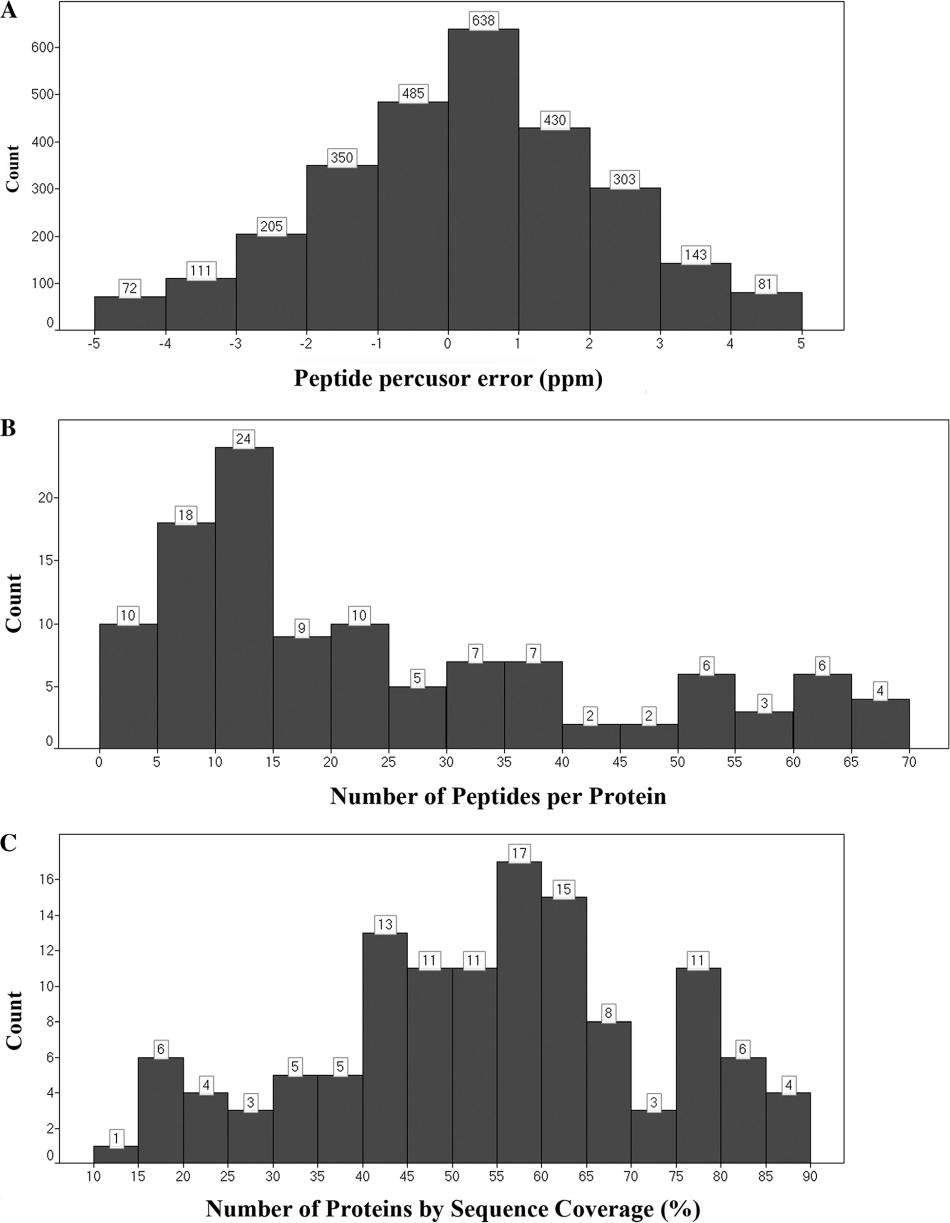
**Experiment PPM error at the peptide level, number of identified peptides per protein and experimental protein sequence coverage.A**) Indicates the number of identified peptides in a 5ppm error range; **B**) Indicates the number of identified peptides per protein; **C**) indicates the number of proteins with sequence coverage from 10 to 90%.

The resulting soybean seed NanoUPLC-MS^E^ peptide data generated by the PLGS process are shown in Figure 1A. The experiment resulted in 3,400 identified peptides; 58% of these peptides were obtained from peptide match type data in the first pass, and 6% were obtained in the second pass [31]. A total of 17% of the peptides were identified by a missed trypsin cleavage, whereas an *in-source* fragmentation rate of 13% was expected for the Synapt G2 data. Figure 2A shows the peptide parts per million error (ppm) indicating that 82% of the peptides were detected with an error of less than 5 ppm. As shown in Figure 2B, 75% of the identified proteins have at least 10 matched peptides, and 88% of the identified proteins have greater than 30% coverage (Figure 2C). The experiment revealed 113 proteins, of which 87% were replicated 4 times, as shown in Figure 1B and Table 1. These results far exceed the minimum protein identification quality compared to other proteomic data, such as those obtained from the 2DE technique, in which only 10 to 20% of the identified proteins exhibit a coverage greater than 30% [14,20].

**Table 1:** Protein identification table

**Uniprot code**	**Description**	**Protein MW**	**ScoreAVG**	**ProductsAVG**	**PeptidesAVG**	**Fmol Covariance**	**NgramAVG**	**Repeate Rate**	**% of TSP**
**P04776**	Glycinin G1 OS Glycine max GN GY1 PE 1 S	56333.71	147104.4	523.25	38.5	0.02	85.28	4	17.68
**Q549Z4**	Proglycinin A2B1 OS Glycine max PE 2 SV	54961.11	69391.44	427.25	38.25	0.67	55.32	4	11.47
**Q4LER5**	Beta conglycinin alpha subunit Fragment	70348.96	105347.27	301.75	54.25	0.75	32.68	4	6.78
**Q3V5S6**	Beta conglycinin alpha subunit OS Glycin	70569.34	104417.56	286.5	53.75	0.72	30.91	4	6.41
**P04405**	Glycinin G2 OS Glycine max GN Gy2 PE 1 S	54961.11	63666.28	370	36.5	1.97	19.12	4	3.96
**Q7XXT2**	Prepro beta conglycinin alpha prime subu	72532.23	28368.36	295	66	0.67	18.17	4	3.77
**P04347**	Glycinin OS Glycine max PE 1 SV 1	58412.39	23431.64	191.5	53	0.22	15.99	4	3.31
**P02858**	Glycinin G4 OS Glycine max GN GY4 PE 1 S	64043.42	31521.33	290.75	60	0.26	14.25	4	2.95
**Q9SB12**	Glycinin OS Glycine max PE 3 SV 1	58685.70	29853.17	251	68.75	0.48	13.30	4	2.76
**Q9S9D0**	Glycinin G4 subunit OS Glycine max PE 3	64135.62	32357.33	309	60.25	0.31	11.44	4	2.37
**Q04672**	Sucrose binding protein OS Glycine max G	60921.87	11679.68	186.25	33.5	0.02	11.37	4	2.36
**Q9ATY1**	Kunitz trypsin inhibitor OS Glycine max	24434.59	35543.40	112.5	13.75	0.17	11.34	4	2.35
**Q948Y0**	Beta conglycinin alpha prime subunit OS	72423.19	17151.17	223.5	65.75	0.75	10.95	4	2.27
**Q948X9**	Beta conglycinin alpha subunit OS Glycin	72760.60	15765.20	186.25	44.25	0.75	9.25	4	1.92
**P05046**	Lectin OS Glycine max GN LE1 PE 1 SV 1	30928.02	10795.06	63.75	10.25	0.01	7.95	4	1.65
**Q94LX2**	Beta conglycinin alpha subunit OS Glycin	70591.40	113386.25	365.5	51.75	1.01	7.57	4	1.57
**P00489**	GLYCOGEN PHOSPHORYLASE MUSCLE FORM EC	97671.64	3801.66	161.5	37.5	0.00	7.33	4	1.52
**P11827**	Beta conglycinin alpha chain OS Glycine	74610.54	11508.88	185.25	65	0.86	7.31	4	1.52
**C6SWW4**	Putative uncharacterized protein OS Glyc	22731.98	17601.57	100.5	12.25	0.07	7.12	4	1.48
**P25974**	Beta conglycinin beta chain OS Glycine m	50609.06	19512.84	186.25	34	0.71	7.10	4	1.47
**Q39898**	Kunitz trypsin inhibitor OS Glycine max	24361.54	54750.93	144.25	14.5	1.16	6.72	4	1.39
**C6T9L1**	Putative uncharacterized protein OS Glyc	50621.07	18189.66	182.5	33.25	0.96	6.30	4	1.31
**P93708**	Glycinin OS Glycine max GN Gly A3B4 PE 2	58691.62	39274.08	237.25	62.5	1.18	5.91	4	1.23
**P13916**	Beta conglycinin alpha chain OS Glycine	70578.31	113406.84	362	49.5	0.76	5.75	4	1.19
**B3TDK4**	Lipoxygenase OS Glycine max PE 3 SV 1	94639.64	4359.21	144.25	35.75	0.03	5.63	4	1.17
**Q93VL9**	Beta conglycinin beta subunit OS Glycine	50580.97	17965.49	159.75	32	0.72	5.29	4	1.10
**Q852U5**	Glycinin A1bB2 445 OS Glycine max PE 2 S	54845.14	19226.30	97	16	0.33	4.59	4	0.95
**P13917**	Basic 7S globulin OS Glycine max GN BG P	47134.49	4532.62	62.5	14	0.03	3.51	4	0.73
**P01070**	Trypsin inhibitor A OS Glycine max GN KT	24290.46	40525.39	125	14	1.97	3.36	4	0.70
**Q70EM0**	Dehydrin OS Glycine max GN lea D 11 PE 3	23787.84	11066.56	101.5	19	0.02	3.24	4	0.67
**Q0MUU5**	Beta conglycinin alpha subunit OS Glycin	70172.28	24742.62	233.5	63.25	2.00	3.14	4	0.65
**C0KG62**	Mutant glycinin A3B4 OS Glycine max PE 2	60494.82	42247.79	242.5	62.25	2.00	2.46	4	0.51
**Q7GC77**	Glycinin A3B4 subunit OS Glycine max PE	58643.62	50693.51	331	57.5	2.00	2.39	4	0.49
**Q53WV6**	Napin type 2S albumin 3 OS Glycine max P	19030.31	21340.11	82.75	13	1.17	2.19	4	0.45
**Q43452**	Glycinin OS Glycine max GN Gy4 PE 3 SV 1	64389.76	32891.71	289.25	50.5	2.00	2.19	4	0.45
**Q852U4**	Glycinin A1bB2 784 OS Glycine max PE 2 S	54868.22	18987.27	81.75	13.5	1.30	1.90	4	0.39
**P10538**	Beta amylase OS Glycine max GN BMY1 PE 1	56485.05	1358.99	57	23.5	0.72	1.87	4	0.39
**Q8RVH5**	Basic 7S globulin 2 OS Glycine max PE 1	47889.37	2086.68	37.25	11.75	0.38	1.87	4	0.39
**Q39853**	Soybean beta conglycinin alpha subunit F	24453.59	88244.12	88.5	9.25	1.89	1.77	4	0.37
**C6SYA7**	Putative uncharacterized protein OS Glyc	19027.27	23176.74	87	15	1.19	1.75	4	0.36
**Q39871**	Late embryongenesis abundant protein OS	50643.91	919.20	40.5	14	0.17	1.65	2	0.34
**C6T7B0**	Putative uncharacterized protein Fragmen	48939.81	23743.45	190.25	54.25	1.16	1.61	4	0.33
**C0J370**	Ribulose bisphosphate carboxylase large	52835.80	2789.04	34.25	9.25	0.75	1.60	4	0.33
**C6TKH0**	Putative uncharacterized protein OS Glyc	32117.31	1246.92	38.75	10	0.03	1.54	4	0.32
**Q9SB11**	Glycinin OS Glycine max GN A5A4B3 PE 2 S	64253.61	38444.74	381.5	49	1.98	1.24	4	0.26
**Q39858**	Soybean glycinin A3 B4 subunit Fragment	27467.17	26110.47	58.5	10.25	2.00	1.20	4	0.25
**Q39816**	7S storage protein alpha subunit OS Glyc	27538.85	8464.43	117.75	29	1.19	0.97	4	0.20
**Q39805**	Dehydrin like protein OS Glycine max PE	23717.75	10257.65	91.5	17	1.61	0.96	4	0.20
**P19594**	2S albumin OS Glycine max PE 1 SV 2	19030.31	20001.72	82	14.75	1.74	0.93	4	0.19
**P11828**	Glycinin G3 OS Glycine max GN GY3 PE 3 S	54869.09	20193.73	96	16.5	0.83	0.86	4	0.18
**C6TDF5**	Putative uncharacterized protein OS Glyc	42166.14	2011.92	45	12.5	0.61	0.81	2	0.17
**Q39876**	Maturation associated protein OS Glycine	23713.76	11376.82	107.75	19.25	1.99	0.80	4	0.17
**Q42795**	Beta amylase OS Glycine max PE 1 SV 1	56413.93	1363.11	54.75	22.5	1.99	0.73	4	0.15
**Q42447**	Maturation protein OS Glycine max GN MAT	25658.99	3286.16	26.25	6.25	0.73	0.64	4	0.13
**Q9SEK9**	Seed maturation protein PM25 OS Glycine	25842.80	1323.54	30.33	7.66	0.37	0.63	3	0.13
**Q9SP11**	Sucrose binding protein homolog S 64 OS	56176.59	2088.61	62.5	24	0.69	0.62	4	0.13
**Q9XET0**	Putative uncharacterized protein OS Glyc	15154.43	2553.76	27.75	5.75	0.03	0.57	4	0.12
**Q9ATY0**	Truncated Kunitz trypsin inhibitor OS Gl	16134.39	18524.95	49.5	7.25	2.00	0.57	4	0.12
**C6T1Q7**	Putative uncharacterized protein OS Glyc	18394.94	4148.8	41.25	10.75	0.67	0.55	4	0.11
**Q9LLQ6**	Seed maturation protein PM34 OS Glycine	32052.25	1013.75	24.5	10	0.39	0.55	2	0.11
**P01064**	Bowman Birk type proteinase inhibitor D	10323.16	4799.89	22.25	4.5	0.68	0.54	4	0.11
**P25273**	Kunitz type trypsin inhibitor KTI2 OS Gl	23085.45	2073.41	19.75	7.25	0.87	0.51	4	0.11
**C6T588**	Putative uncharacterized protein OS Glyc	16817.85	2001.68	23	6.25	0.14	0.49	4	0.10
**A1KR24**	Dehydrin OS Glycine max GN LEA 2 D11 PE	25384.72	3672.93	34.25	8.75	0.44	0.46	4	0.10
**P09439**	Seed lipoxygenase 2 OS Glycine max GN LO	97430.94	763.76	45	27	1.41	0.40	2	0.08
**C6TB67**	Putative uncharacterized protein OS Glyc	23271.56	1060.30	16	5.33	0.03	0.40	3	0.08
**P27066**	Ribulose bisphosphate carboxylase large	53066.07	2905.08	31.75	8.75	2.00	0.37	4	0.08
**P01063**	Bowman Birk type proteinase inhibitor C	9999.68	5359.28	33.25	4.25	0.10	0.34	4	0.07
**B3TDK5**	Lipoxygenase OS Glycine max PE 3 SV 1	97068.50	753.71	44	26.5	1.41	0.33	2	0.07
**Q39875**	Soybean lipoxygenase 1 Fragment OS Glyci	36808.29	1111.12	23	12.333333	1.65	0.31	3	0.06
**Q9SBA9**	Bowman Birk proteinase inhibitor Fragmen	5100.41	8774.56	15	1	0.03	0.31	4	0.06
**Q76B18**	Kunitz trypsin inhibitor OS Glycine max	24433.60	24408.10	91	12.5	2.00	0.30	4	0.06
**O23957**	Dehydrin OS Glycine max GN GmPM12 PE 2 S	17319.94	1858.29	13.5	3	1.41	0.28	2	0.06
**C6T9Z5**	Putative uncharacterized protein OS Glyc	43109.03	1650.38	28	11.5	1.41	0.27	2	0.06
**Q84V19**	Sucrose binding protein 2 OS Glycine max	56117.52	2226.42	65.75	23.25	1.93	0.25	4	0.05
**O22121**	Beta subunit of beta conglycinin Fragmen	47975.72	20300.60	200.5	32.75	2.00	0.25	4	0.05
**P08170**	Seed lipoxygenase 1 OS Glycine max GN LO	94597.61	3028.73	90.75	35.75	1.18	0.21	4	0.04
**Q53B72**	Putative chalcone isomerase 4 OS Glycine	23495.71	1460.83	11.5	2.5	0.01	0.20	2	0.04
**Q43709**	Bowman Birk proteinase isoinhibitor D II	12351.73	5458.41	27	4.5	2.00	0.20	4	0.04
**Q9ZNZ4**	Napin type 2S albumin 1 OS Glycine max P	18404.98	4001.44	39.75	10.5	2.00	0.18	4	0.04
**Q94IA1**	Kunitz trypsin inhibitor OS Glycine max	24333.52	34662.18	107	12	2.00	0.18	4	0.04
**Q588Z3**	Beta amylase OS Glycine max GN Gm BamyDa	56441.98	1367.07	58.5	22.75	2.00	0.16	4	0.03
**C7EA91**	Mutant glycinin subunit A1aB1b OS Glycin	44092.82	71745.98	326.25	33	0.42	0.15	4	0.03
**Q70EL8**	Dehydrin OS Glycine max GN lea D 11 PE 4	23733.70	9455.43	82.25	16.5	2.00	0.11	4	0.02
**P93165**	Em protein OS Glycine max PE 4 SV 1	11491.34	3266.78	22.25	6	0.06	0.11	4	0.02
**C6TBB3**	Putative uncharacterized protein OS Glyc	12345.51	1829.78	14.75	4.5	0.21	0.10	4	0.02
**Q4LER6**	Beta conglycinin alpha prime subunit OS	72513.18	26588.66	264.25	63.5	2.00	0.09	4	0.02
**Q7M1N5**	Glycinin A1aB1b Fragments OS Glycine max	6315.28	13404.83	14.5	3.5	1.09	0.07	4	0.01
**P25272**	Kunitz type trypsin inhibitor KTI1 OS Gl	22831.11	11727.02	70.75	12.5	0.16	0.06	4	0.01
**C7EA92**	Mutant glycinin subunit A1aB1b OS Glycin	43990.73	65306.21	275.25	30	0.70	0.05	4	0.01
**C6T7Y4**	Putative uncharacterized protein OS Glyc	33364.68	1386.46	32.5	13.5	2.00	0.04	4	0.01
**Q5K3Q9**	Putative dehydrin Fragment OS Glycine ma	20395.17	7625.21	70.5	15.75	2.00	0.00	4	0.00
**P01071**	Trypsin inhibitor B OS Glycine max PE 1	20268.75	17312.94	48.75	7.5	2.00	0.00	4	0.00
**Q9FZP9**	Alpha subunit of beta conglycinin Fragme	65199.76	25899.30	239.25	55.5		0.00	4	0.00
**O22120**	Alpha subunit of beta conglycinin Fragme	63221.91	105305.79	299.75	42.5		0.00	4	0.00
**Q588Z5**	Beta amylase OS Glycine max GN Gm BamyKz	56419.97	1193.51	46.25	20.75		0.00	4	0.00
**Q588Z6**	Beta amylase OS Glycine max GN Gm BamyTk	56499.03	945.94	42	20		0.00	4	0.00
**Q588Z4**	Beta amylase OS Glycine max GN Gm BamyTk	56426.96	1290.96	50.5	23.25		0.00	4	0.00
**Q45UE7**	Beta amylase OS Glycine max PE 2 SV 1	56412.98	1420.14	54.25	21.25		0.00	4	0.00
**Q84UB3**	Beta conglycinin alpha subunit Fragment	45018.62	24184.62	193.75	39.75		0.00	4	0.00
**Q50JD8**	Beta conglycinin beta subunit Fragment O	48387.22	22348.6	219	28.25		0.00	4	0.00
**Q70EL7**	Dehydrin OS Glycine max GN lea D 11 PE 3	25275.54	3943.08	30	8		0.00	3	0.00
**Q70EL9**	Dehydrin OS Glycine max GN lea D 11 PE 3	25551.97	3591.29	24	5.6666665		0.00	3	0.00
**Q7M211**	Glycinin A3B4 Plasmid pSPGD41 Fragment O	21495.15	29529.21	86	16.75		0.00	4	0.00
**Q7M210**	Glycinin A3B4 Plasmid pSPGL1 Fragment OS	27293.87	30284.32	105.25	23.75		0.00	4	0.00
**Q6LBP7**	Glycinin B 1b subunit 15 AA Fragment OS	1698.00	8521.25	7.6666665	2.3333333		0.00	3	0.00
**P93707**	Glycinin OS Glycine max GN Gly A3B4 PE 2	58633.58	45602.53	283	58.75		0.00	4	0.00
**Q9LD16**	Kunitz trypsin inhibitor 3 OS Glycine ma	24295.44	27398.43	66.25	9.25		0.00	4	0.00
**Q39899**	Kunitz trypsin inhibitor OS Glycine max	24308.45	17312.92	48.75	7.5		0.00	4	0.00
**Q7XAW0**	Lea protein OS Glycine max GN ZLDE 2 PE	25368.76	3593.48	33.75	8.5		0.00	4	0.00
**Q39870**	Lipoxygenase OS Glycine max GN lox2 PE 2	97551.04	754.83	39	25.5		0.00	2	0.00
**C6T488**	Putative uncharacterized protein OS Glyc	24403.62	35506	113.5	13.5		0.00	4	0.00
**C6SX26**	Putative uncharacterized protein OS Glyc	12914.35	3157.77	23.5	3.5		0.00	4	0.00

The ScoreAVG is the average PLGS score for each hit. ProductsAVG is the average fragment ion products for a protein hit. PeptideAVG is the average of the peptide hits per identified protein. FmolCovariance is the covariance at the femtomole detection level for each protein in the replicate analyses. NgramAVG is the average (in nanograms) of each protein load on the column. The repetition rate is the repeatability of each protein in the replicates. % of TSP is the percentage of each protein in the total soluble mixture relative to the total protein load (in nanograms) on the column.

**Figure 3:**
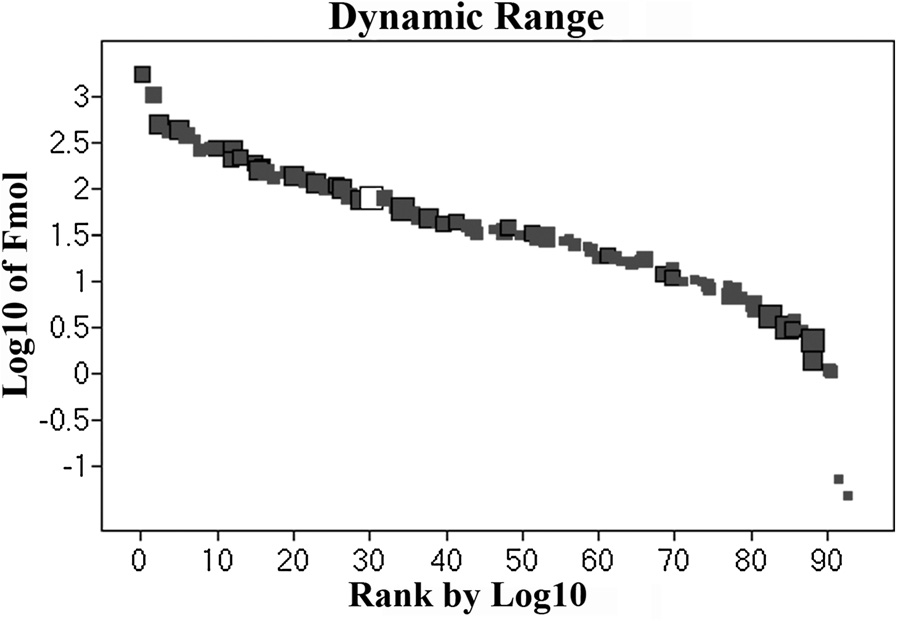
**Detection dynamic range of the experiment.** White square represent rabbit phosphorylase B as an external standard. Dark squares indicate proteins identified in the experiment. The size of each square represents the molecular weight of the identified protein.

Figure 3 shows the results obtained from dynamic range detection, indicating that 95 proteins were quantified. A 3-log range and a good detection distribution of high and low molecular weights were obtained, as indicated by the size of the squares. The 10 least abundant proteins includes B3TDK5_SOYBN-Lipoxygenase, C7EA91_SOYBN-Mutant glycinin subunit A1aB1b, Q588Z3_SOYBN-Beta amylase, KTI1_SOYBN-Kunitz type trypsin inhibitor, LOX1_SOYBN-Seed lipoxygenase 1, Q4LER6_SOYBN-Beta conglycinin alpha prime, C7EA92_SOYBN-Mutant glycinin subunit A1aB1b, C6T7Y4_SOYBN-Putative uncharacterized protein, Q5K3Q9_SOYBN-Putative dehydrin Fragment and ITRB_SOYBN-Trypsin inhibitor B. The 10 most abundant proteins are composed of GLYG1_SOYBN-Glycinin G1, Q549Z4_SOYBN-Proglycinin A2B1, Q4LER5_SOYBN-Beta conglycinin alpha subunit, Q9ATY1_SOYBN-Kunitz trypsin inhibitor, Q3V5S6_SOYBN-Beta conglycinin alpha subunit, GLYG2_SOYBN-Glycinin G2, C6SWW4_SOYBN-Putative uncharacterized protein, Q39898_SOYBN-Kunitz trypsin inhibitor, GLYG5_SOYBN-Glycinin and LEC_SOYBN-Lectin. A detailed description of each protein is presented in Table 1. A comparison of our results with other proteomic data reveals that there is a discrepancy in the number of proteins that were identified. Barbosa et al. [14] described 192 identified proteins, although these 192 2DE spots likely correspond to a lower number of proteins because many of the identifications are associated with the same protein with a pI shift. The same trend can be observed in the work presented by Mooney et al. [20], which described 96 identifications of 150 spots detected via 2DE. Sakata et al. [32] described more than 500 spots in gels from cotyledons but reported only 34 identified proteins. Our results mainly identify single proteins. However, there were some exceptions, especially for proteins that possess subunits with similar amino acid sequences, such as glycinin and beta-conglycinin, but are identified with a different accession number in the Uniprot database.

We also compared the identified proteins and correlated them to their function. All identified proteins having at least two replicates are shown in Table 1. A total of 42% of these data correspond to storage proteins, as shown in Figure 1C. From TSP, 78% of the identified species correspond to glycinin and beta-conglycinin chains: GLYG1_SOYBN-Glycinin and Q549Z4_SOYBN-Proglycinin A2B1 correspond to 18% and 12% of TSP, respectively [33]; 13% correspond to inhibitors, including Q9ATY1_SOYBN kunitz type (2.35% of TSP) and Q9SBA9_SOYBN Bowman Birk proteinase inhibitor (0.06% of TSP) [34]; 16% correspond to energy-related proteins, such as C0J370_SOYBN Ribolose (0.33% TSP), Q42795_SOYBN beta-amylases (0.57%) and B3TDK4_SOYBN lipoxygenase (1.14% TSP); and 17% are associated with abiotic stress, including Q7XAW0_SOYBN dehydrin (1% TSP) and LEA proteins (<0.01 % TSP) [35]. An additional 13% correspond to putative uncharacterized proteins. Late embryogenesis proteins and maturation proteins represent 7% of the proteins, including Q39871_SOYBN (0.34% TSP), P93165_SOYBN Em protein (0.2% TSP) and Q9LLQ6_SOYBN seed maturation protein (0.13% TSP) [36,37]. These results are in agreement with those of other studies [15,17,20,32].

### Uniprot data assessment

**Figure 4:**
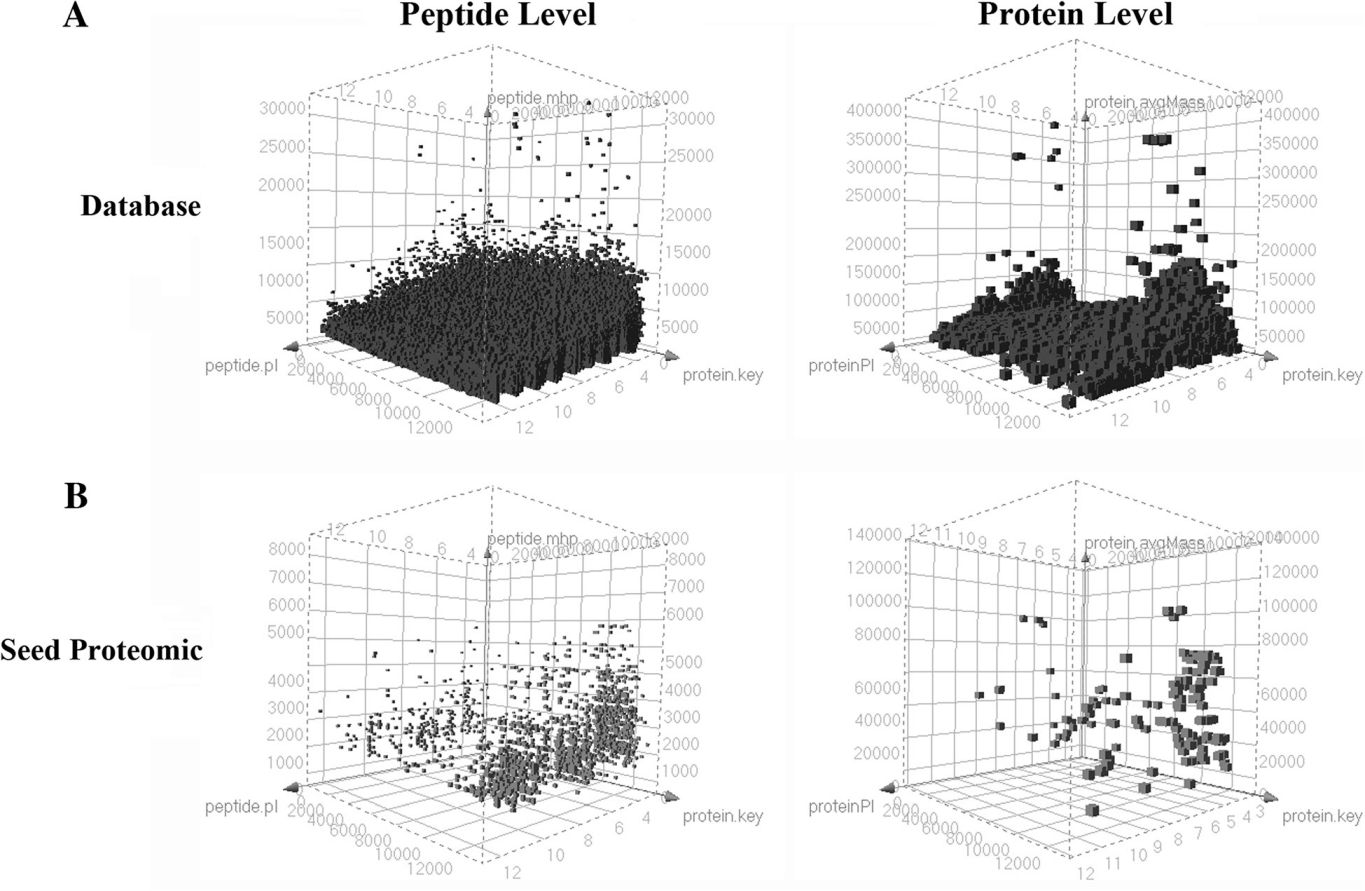
**Distribution of peptides and proteins in the Uniprot database and seed proteomics by NanoUPLC-MS**^**E**^**.A**) Corresponds to the database proteins; **B**) Indicates the proteins identified in this work.

**Figure 5:**
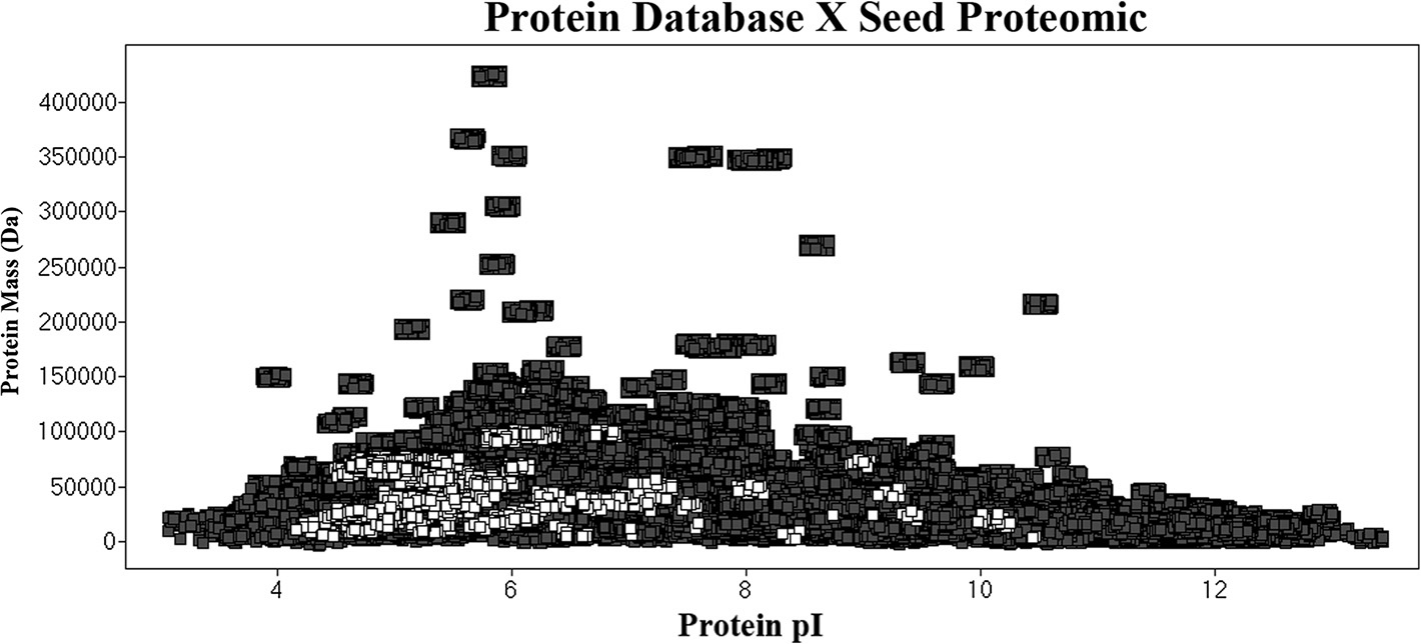
**Distribution of seed proteins identified within the Uniprot protein database by the isoelectric point.** Data shown in dark squares correspond to database proteins, and data shown in white squares are hits identified by this work.

**Figure 6:**
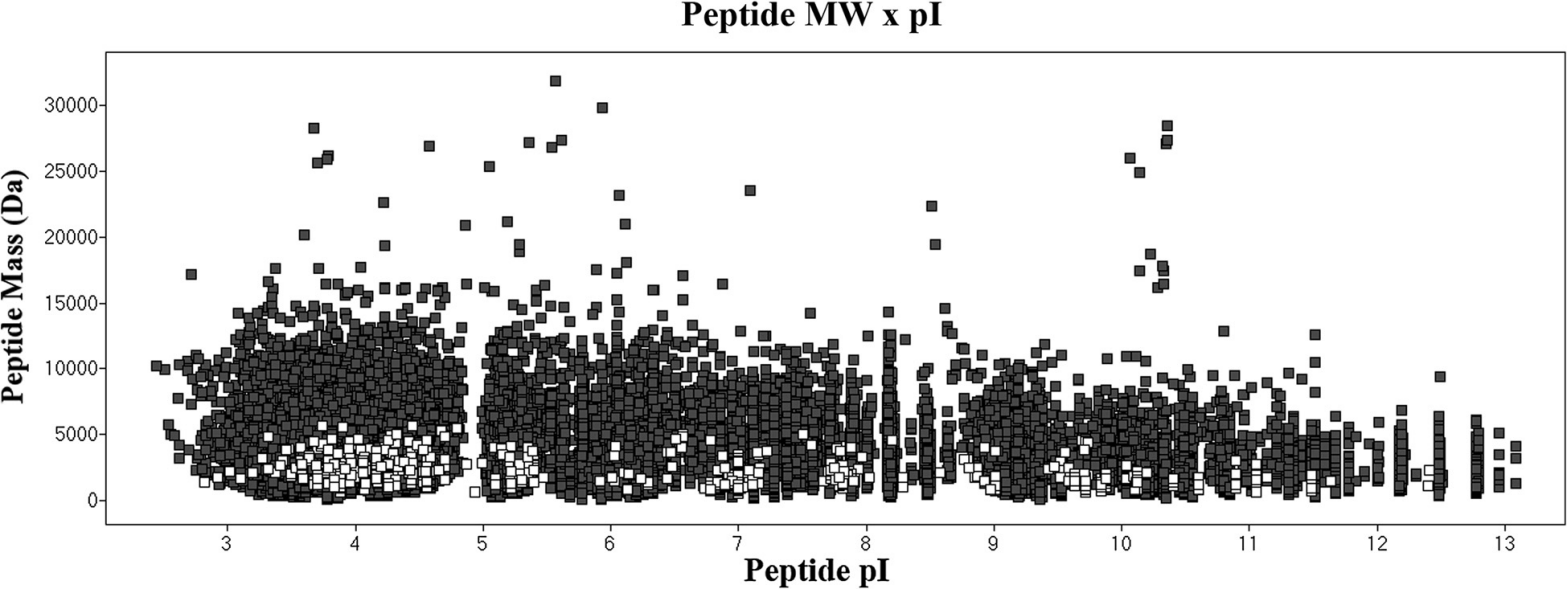
**Distribution of peptide isoelectric points for the database and identified peptides.** Dark squares corresponds to database peptides. White squares represents peptides identified using NanoUPLC-MS^E^.

There are 13,117 soybean sequence entries in the Uniprot database. The theoretical tryptic digestion results show 368,435 peptides. Assuming one missed cleavage, the theoretical peptide database has 723,749 entries, 548,336 of which possess a molecular weight greater than 500 Da. These results and a comparison with proteomic data are presented in Figure 4. Seed proteins represent 0.86% of the protein database entries. At the peptide level, trypsin-digested seed proteins represent only 0.3% of the theoretical peptide database, including missed cleavage proteins, which are responsible for only 0.08% of the identified data. Of the seed proteins detected in our experiments, 78% have a pI value between 4.2 and 6. This result is presented in Figure 5, which shows that seed proteins have acidic characteristics. This characteristic was also reported by Robic et al. [38]. At the peptide level (Figure 6), 22% of all database peptides have a pI of less than 5, and 25% of them have a pI between 5 and 8. Figure 6 also shows that 43% of peptides resulting from the experiments have a pI value of less than 5. This pattern is characteristic of tryptic digestion and LC-ESI-MS experiments because the method favors charged peptides.

In Table 2, we present the number of proteins that are not detected within a particular peptide molecular mass detection range. When assuming the minimum and maximum peptide detection levels found using NanoUPLC-MS^E^ experiments, i.e., 500 to 5000 Da, 64 proteins do not have detectable peptides after trypsin digestion (Table 2 and 3). The majority of these proteins correspond to putative and uncharacterized proteins, although NU6C_SOYBN NAD(P)H-quinone oxidoreductase is within the detection range and is not related to seed proteins. Assuming 1 peptide at a threshold of 5,000 Da (Table 3), a few seed proteins are not detected by NanoUPLC-MS^E^: ACT6_SOYBN Actin-6, ACT7_SOYBN Actin-7, ALL50_SOYBN Major Gly 50 kDa allergen and Q7M212_SOYBN Water-soluble 35K protein. With 2 peptides at an upper threshold of 5,000 Da (Table 3), several putative proteins are not detected, including Q3HM31_SOYBN Hydrophobic seed protein and Q692Y3_SOYBN Glycinin gy1 (Fragment). With 3 peptides at an upper threshold of 5,000 Da (Table 3), the not detected protein list is mainly composed of putative and uncharacterized proteins and other protein fragments that have short amino acid sequences.

**Table 2:** Number of proteins with 0 peptides over a given peptide detection range

**Peptide MW range**	**Number of proteins that has 0 peptides**
**500<x<1000**	379
**500<x<2000**	139
**500<x<3000**	101
**500<x<4000**	73
**500<x<5000**	64
**1000<x<2000**	311
**2000<x<3000**	700
**3000<x<4000**	2163
**4000<x<5000**	4820
**5000<x<6000**	7953
**>6000**	9006

**Table 3:** Uniprot access codes for proteins containing 0, 1, 2 or 3 peptides for a given database peptide detection threshold

**Nº**	**500<x<5000 MW cutoff**				**500<x<3000 MW cutoff**			
	**0.peptides**	**1.peptides**	**2.peptides**	**3.peptides**	**0 peptides**	**1.peptides**	**2.peptides**	**3.peptides**
**1**	C6T0N1	A8ICX6	B3TDL0	C6SWN8	C6SZL6	A8ICX6	B3TDL0	B4XH43
**2**	C6T0N3	A8IFI6	C6SV77	C6SYE8	C6T029	A8IFI6	C6SV77	C6SVW9
**3**	C6T0N7	C6SV73	C6SVQ7	C6SYW9	C6T0N1	C6SV73	C6SVK4	C6SVX8
**4**	C6T0R2	C6SW74	C6SWW3	C6SZA0	C6T0N3	C6SW74	C6SVQ7	C6SZD7
**5**	C6T0R6	C6SXX1	C6SXB8	C6T0K8	C6T0N7	C6SWU9	C6SWN8	C6SZQ4
**6**	C6T0S2	C6SY49	C6SY76	C6T0W0	C6T0Q2	C6SXX1	C6SWW3	C6SZT3
**7**	C6T0S5	C6SYJ1	C6SYR8	C6T0Y8	C6T0R2	C6SY49	C6SX62	C6SZT6
**8**	C6T0T0	C6T079	C6SYR9	C6T2N3	C6T0R6	C6SYJ1	C6SXB8	C6T013
**9**	C6T0T6	C6T0X6	C6SZK8	C6T4B2	C6T0S2	C6SYR8	C6SXL1	C6T022
**10**	C6T0U0	C6T113	C6SZV3	C6T5Y8	C6T0S5	C6SYW9	C6SY37	C6T0H4
**11**	C6T0U6	C6T128	C6T0P3	C6T604	C6T0T0	C6SZA0	C6SY76	C6T0W0
**12**	C6T0U8	C6T145	C6T0P7	C6T695	C6T0T6	C6SZK8	C6SYE8	C6T0Y8
**13**	C6T0V0	C6T149	C6T0Q2	C6T697	C6T0U0	C6SZM6	C6SYI5	C6T196
**14**	C6T0V2	C6T5D7	C6T0R0	C6T6A1	C6T0U6	C6T079	C6SYL8	C6T1C2
**15**	C6T0V4	C6T5W7	C6T0R4	C6T6D9	C6T0U8	C6T0K8	C6SYR9	C6T1C3
**16**	C6T0V8	C6T614	C6T0R8	C6T6I0	C6T0V0	C6T0X6	C6SZV3	C6T1N5
**17**	C6T0W6	C6T635	C6T0S8	C6T6I2	C6T0V2	C6T115	C6T0P3	C6T272
**18**	C6T0W8	C6T656	C6T0T4	C6T6J6	C6T0V4	C6T145	C6T0P7	C6T2A4
**19**	C6T0Y0	C6T676	C6T0W2	C6T6Q9	C6T0V8	C6T149	C6T0R0	C6T343
**20**	C6T108	C6T6A8	C6T0W4	C6T6S9	C6T0W6	C6T173	C6T0R4	C6T382
**21**	C6T110	C6T6C5	C6T0X2	C6T6T3	C6T0W8	C6T1V9	C6T0R8	C6T384
**22**	C6T124	C6T6D5	C6T0X4	C6T6U7	C6T0X2	C6T3J3	C6T0S8	C6T400
**23**	C6T130	C6T6F4	C6T0Y2	C6T6U8	C6T0Y0	C6T426	C6T0T4	C6T4D2
**24**	C6T132	C6T6H2	C6T0Y4	C6T7A3	C6T108	C6T4U9	C6T0V6	C6T4E9
**25**	C6T137	C6T6L2	C6T0Z0	C6TDF1	C6T110	C6T5D7	C6T0W2	C6T4I4
**26**	C6T141	C6T6L9	C6T0Z5	C6TE34	C6T113	C6T5Y5	C6T0W4	C6T4U1
**27**	C6T5Q9	C6T6R9	C6T0Z7	C6TFK5	C6T124	C6T614	C6T0X4	C6T5Y9
**28**	C6T5V9	C6T6V2	C6T0Z9	C6TFK6	C6T128	C6T635	C6T0Y2	C6T5Z3
**29**	C6T5W3	C6T6X9	C6T101	C6TFL5	C6T130	C6T656	C6T0Y4	C6T604
**30**	C6T602	C6T6Y2	C6T122	C6TFS1	C6T132	C6T676	C6T0Z0	C6T661
**31**	C6T606	C6T7V8	C6T143	C6TFT9	C6T137	C6T6A8	C6T0Z5	C6T695
**32**	C6T618	C6TC31	C6T1V9	C6TGK1	C6T141	C6T6C5	C6T0Z7	C6T6A1
**33**	C6T620	C6TE40	C6T2B3	C6THI2	C6T2D4	C6T6F4	C6T0Z9	C6T6B5
**34**	C6T643	C6TG02	C6T2D4	C6TIA8	C6T2Q5	C6T6H2	C6T101	C6T6I9
**35**	C6T664	C6TGX9	C6T2Q5	C6TIG4	C6T5N2	C6T6L2	C6T122	C6T6K7
**36**	C6T666	C6TIG9	C6T2Z6	C6TKN2	C6T5Q9	C6T6L9	C6T143	C6T6M6
**37**	C6T684	C6TKR3	C6T3C3	C6TMR8	C6T5U2	C6T6R9	C6T144	C6T6Q9
**38**	C6T688	C6TLP1	C6T5U2	C6TNC0	C6T5U9	C6T6V2	C6T233	C6T6T3
**39**	C6T6C7	C6TN83	C6T5U9	C6TNW8	C6T5V9	C6T6Y2	C6T269	C6T6U7
**40**	C6T6E5	O49223	C6T5V1	O49225	C6T5W3	C6T774	C6T2B3	C6T6U8
**41**	C6T6F5	P13993	C6T5X3	P08012	C6T5W7	C6T7U7	C6T2N3	C6T6W7
**42**	C6T6G1	P15986	C6T5Y1	P08297	C6T5Y1	C6TA13	C6T2Z6	C6T6X5
**43**	C6T6H7	P15987	C6T5Z8	P49159	C6T5Z8	C6TB42	C6T3A7	C6T743
**44**	C6T6J2	P55960	C6T608	Q2PMQ7	C6T602	C6TD14	C6T3C3	C6T7A3
**45**	C6T6J4	P82947	C6T622	Q2PMR6	C6T606	C6TD15	C6T404	C6T7H3
**46**	C6T6J8	Q6JC67	C6T629	Q2PMR9	C6T618	C6TDF1	C6T411	C6T8U0
**47**	C6T6J9	Q6JC68	C6T631	Q2PMS0	C6T620	C6TDT6	C6T4B2	C6T9A8
**48**	C6T6K2	Q7M212	C6T633	Q2PMT7	C6T643	C6TE71	C6T5V1	C6T9K4
**49**	C6T6L0	Q84U84	C6T639	Q39829	C6T652	C6TG02	C6T5X3	C6TA51
**50**	C6T6L7	Q8S3C5	C6T645	Q4W663	C6T664	C6TGS5	C6T5Y8	C6TAK0
**51**	C6T6N6	Q8S3C6	C6T649	Q6LBP7	C6T666	C6TIG4	C6T608	C6TBL2
**52**	C6T6Q0	Q8W238	C6T652	Q6Q0T0	C6T671	C6TIG9	C6T622	C6TDM5
**53**	C6T6Q2	Q9JMW3	C6T659	Q6X0N6	C6T678	C6TKR3	C6T629	C6TDP4
**54**	C6T6Q6	Q9S8F2	C6T671	Q7M1K4	C6T684	C6TL18	C6T631	C6TDP6
**55**	C6T6S4	Q9S8F3	C6T678	Q9S8R7	C6T688	C6TL51	C6T633	C6TDR0
**56**	C6T6T1	Q9S8K3	C6T682	Q9S8X5	C6T6C7	C6TLP1	C6T639	C6TDR8
**57**	C6T6T7	Q9S904	C6T686	Q9S926	C6T6D5	O49223	C6T645	C6TEN4
**58**	C6T6T9	Q9S905	C6T690	Q9SBB0	C6T6E5	P13993	C6T649	C6TF71
**59**	C6T6W6	Q9S929	C6T693		C6T6F5	P15986	C6T659	C6TFK5
**60**	C6T6W8				C6T6G1	P15987	C6T682	C6TFS1
**61**	C6T837				C6T6H7	P49159	C6T686	C6TFT9
**62**	C6TDZ9				C6T6I0	P55960	C6T690	C6TFY8
**63**	C6TN18				C6T6J2	P82947	C6T693	C6TGK1
**64**	Q2PMN5				C6T6J4	Q2PMQ7	C6T697	C6TGZ6
**65**					C6T6J8	Q2PMS0	C6T698	C6TIK8
**66**					C6T6J9	Q2PMS6	C6T6B0	C6TJX4
**67**					C6T6K2	Q2PMT6	C6T6B7	C6TKH6
**68**					C6T6K4	Q2PMT7	C6T6D9	C6TKN2
**69**					C6T6L0	Q2PMU0	C6T6F3	C6TLE1
**70**					C6T6L7	Q39806	C6T6F7	C6TLT6
**71**					C6T6N6	Q4W663	C6T6H9	C6TLV5
**72**					C6T6P1	Q6JC67	C6T6I2	C6TM33
**73**					C6T6Q0	Q6JC68	C6T6J6	C6TM52
**74**					C6T6Q2	Q6X0N6	C6T6K0	C7S8C3
**75**					C6T6Q6	Q7M212	C6T6K6	O49225
**76**					C6T6S4	Q84U84	C6T6R3	O65110
**77**					C6T6T1	Q8S3C6	C6T6S9	P08012
**78**					C6T6T7	Q8W238	C6T6W1	P69421
**79**					C6T6T9	Q9JMW3	C6T737	Q2PMQ8
**80**					C6T6W6	Q9S8K3	C6T7G8	Q2PMR4
**81**					C6T6W8	Q9S904	C6T7P1	Q2PMS7
**82**					C6T6X9	Q9S905	C6T7Y0	Q39829
**83**					C6T7V8	Q9S929	C6T9H9	Q39863
**84**					C6T837		C6TBB6	Q41267
**85**					C6TC31		C6TBR9	Q4W666
**86**					C6TCV3		C6TBS2	Q6J5U8
**87**					C6TDZ9		C6TBV4	Q6LBP7
**88**					C6TE40		C6TD51	Q6LED6
**89**					C6TFL5		C6TD69	Q7M1K4
**90**					C6TGX9		C6TE34	Q9S8R7
**91**					C6TLD5		C6TEN7	Q9S8X5
**92**					C6TLV0		C6TF61	Q9S926
**93**					C6TN00		C6TF86	Q9S9H5
**94**					C6TN18		C6TFK6	
**95**					C6TN83		C6TFW2	
**96**					Q2PMN5		C6TFY6	
**97**					Q3HM31		C6TH40	
**98**					Q8S3C5		C6THI2	
**99**					Q9S8F2		C6TIA8	
**100**					Q9S8F3		C6TJW9	
**101**					Q9SBB0		C6TK75	
**102**							C6TKY5	
**103**							C6TLF7	
**104**							C6TMR8	
**105**							C6TNC0	
**106**							C6TNG5	
**107**							C6TNW8	
**108**							O65109	
**109**							P08297	
**110**							P69195	
**111**							Q0GPJ4	
**112**							Q2PMR5	
**113**							Q2PMR6	
**114**							Q2PMR9	
**115**							Q2TI80	
**116**							Q4W664	
**117**							Q692Y3	
**118**							Q6J5X9	
**119**							Q6Q0T0	
**120**							Q75NI2	
**121**							Q7M285	
**122**							Q9S8H6	
**123**							Q9S8P7	
**124**							Q9S8X4	
**125**							Q9S8X6	

Many of these undetected proteins have been found in soybean seeds and described in other studies [15,17,20,32]. An analysis of the undetected proteins in the database shows that the majority of the sequences are composed of short amino acid sequences with at most 20 residues. This observation may explain the level of missed detection in the NanoUPLC experiments. Other proteins that are not described in this work, such as glyceraldehyde 3-phosphate (Q2I0H4_SOYBN), Malate dehydrogenase (B0M1B0_SOYBN), Glutathione S-transferase (C6ZQJ7_SOYBN), Isoflavone reductase (Q9SDZ0_SOYBN), Alcohol dehydrogenase 1 (Q8LJR2_SOYBN) and In2-1 protein (Q9FQ95_SOYBN), have been described as soybean seed proteins. These proteins have been described in a previous study on the proteomics of transgenic soybean seeds expressing CTAG recombinant proteins [23]. Further experiments must be performed to clarify this issue.

We hypothesize that environmental stress may have altered the seed expression profiles because the EMBRAPA BR-16 seeds were cultivated in the field, and the transgenic seeds were grown in a greenhouse. For example, Barbosa et al. [14] and Brandão et al. [22] reported different expression levels of enzymes in the transgenic soybean proteome of Monsanto Roundup-ready seeds. The authors state that the genetic modification itself could be a stress factor and may produce alterations in the seed proteome. A comparison between the results of this work and our previous study provides evidence in support of this hypothesis, indicating the need for further experiments to confirm possible proteome alterations due to genetic modification. Nevertheless, highly hydrophobic or insoluble proteins will not be detected due to the necessity for in-solution protease digestion; special protocols are needed for the digestion of these types of protein.

## Conclusions

NanoUPLC-MS^E^ experiments are a viable choice as a proteomic pipeline for soybean protein detection. NanoUPLC-MS^E^ provides good protein coverage with a 5 ppm peptide error, reduced sample manipulation relative to other techniques and detection of a wide range of peptide MWs, i.e., from 500 to 5000 Da. Because not all proteins from the Uniprot database are covered, the use of a second digestion enzyme is recommended depending on the tissue to be analyzed. In the case of seed tissue, trypsin protein digestion results in good database coverage. The Uniprot database has many duplicate entries that may result in false protein homolog association and must be formatted prior to use or the use of the reviewed sequences only. It also has many fragment entries that are not suitable for NanoUPLC-MS^E^ analysis but may be used in other techniques. The proteomic profile of EMBRAPA BR-16 seed lacks certain described proteins relative to transgenic soybean profiles reported in other studies. This discrepancy demonstrates the need for further transgenic and nontransgenic proteome analyses.

## Methods

### Extraction of total soluble protein from soybean seeds

Seeds from the EMBRAPA BR-16 cultivar were used in this work. The soybean seeds were ground to a fine powder using a coffee grinder. A 100 mg sample of powder was weighed and placed in a 2 mL capped centrifuge tube. Petroleum ether (1 mL) was added, and the sample was gently agitated for 15 min. The supernatant was discarded, and this step was repeated twice. The petroleum ether was evaporated for 10 min, and 1 mL of 20 mM Tris–HCl pH 8.3, 1.5 mM KCl, 10 mM DTT, 1 mM PMSF and 0.1% V/V SDS was added. The sample was slowly vortexed at room temperature for 10 min and centrifuged for 5 min at 10000g at 4°C. The supernatant was then transferred to a new centrifuge tube. For each 200 μL of sample, 800 μL of cold acetone was added to the centrifuge tube. The sample was vortexed thoroughly and incubated at −20°C for 1 h with vortexing performed every 15 min. The sample was then centrifuged for 10min at 15700g. The supernatant was discarded, and the pellet was dried at room temperature for 30min. The pellet was carefully dissolved in 500 μL of 50 mM ammonium bicarbonate and quantified using a Quant-iT™ Protein Assay Kit (Invitrogen, USA). The sample was finally diluted with 50 mM ammonium bicarbonate to a protein concentration of 1 μg.μL^-1^.

### Sample preparation for NanoUPLC-MS^E^ acquisition

A 50 μL aliquot of the 1 μg.μL^-1^ sample was added to 10 μL of 50 mM ammonium bicarbonate in a microcentrifuge tube. Then, 25 μL of RapiGEST™ (Waters, USA) (0.2% v/v) was added, and the sample was vortexed and incubated in a dry bath at 80°C for 15 min. The sample was briefly centrifuged, and 2.5 μL of 100 mM DTT was added. The sample was vortexed gently and incubated at 60°C for 30 min followed by centrifugation. Iodoacetamide (2.5 μL of a 300 mM solution) was added, and the sample was briefly vortexed and incubated in the dark at room temperature for 30 min. Then, 10 μL of trypsin (with 400 μL of 50 mM ammonium bicarbonate added per 20 μg vial of trypsin) was added, and the sample was briefly vortexed. The sample was digested at 37°C in a dry bath overnight. To cleave and precipitate the RapiGEST™, 10 μL of a 5% TFA solution was added, and the sample was vortexed, incubated for 90 min at 37°C in a dry bath, and centrifuged at 18000g at 6°C for 30 min. The supernatant was transferred to a Waters Total Recovery vial (Waters, USA), and 5 μL of Rabbit Phosphorylase B (Waters, part number 186002326) (with 1 mL of 3% acetonitrile and 0.1% formic acid) and 85 μL of a 3% acetonitrile and 0.1% formic acid solution were added. The final concentration of the protein was 250 ng.μL^-1^, and the final concentration of Phosphorylase B was 25 fmol.μL^-1^. The final volume was 200 μL.

### NanoUPLC-MS^E^ acquisition

The nanoscale LC separation of tryptic peptides from TSP was performed using a nanoACQUITY™ system (Waters Corp., USA) equipped with a Symmetry C18 5μm, 5mm x 300μm precolumn and a nanoEase™ BEH130 C18 1.7 μm, 100 μm x 100 mm analytical reversed-phase column (Waters, USA). The samples were initially transferred to the pre-column using an aqueous 0.1% formic acid solution with a flow rate of 5 μL.min^-1^ for 2 min. Mobile phase A consisted of 0.1% formic acid in water, and mobile phase B consisted of 0.1% formic acid in acetonitrile. The peptides were separated using a gradient of 3-40% mobile phase B for 200 min with a flow rate of 600 ηL.min^-1^ followed by a 10 min rinse with 85% of mobile phase B. The column was re-equilibrated to the initial conditions for 20 min. The column temperature was maintained at 35°C. The lock mass was delivered from the fluidics system of a SynaptG2 pump using a constant flow rate of 400 ηL.min^-1^ at a concentration of 200 fmol of GFP to the reference sprayer of the NanoLockSpray source of the mass spectrometer. All samples were analyzed in four replicates.

The tryptic peptides were analyzed using a Synapt G2 HDMS™ mass spectrometer (Waters, Manchester, UK) with a hybrid quadrupole/ion mobility/orthogonal acceleration time-of-flight (oa-TOF) geometry. For all measurements, the mass spectrometer was operated in the sensitive mode of analysis with a typical resolving power of at least 10000 full-width half-maximum (FWHM). All analyses were performed using a positive nanoelectrospray ion mode (nanoESI +). The time-of-flight analyzer of the mass spectrometer was externally calibrated with GFP b+ and y+ ions from 50 to 1990 m/z with the data post acquisition lock mass corrected using the GFP double charged precursor ion [M + 2H]^2+^ = 785.8426. The reference sprayer was sampled at a frequency of 30 s. The exact mass retention time (EMRT) [28] nanoLC-MS^E^ data were collected in an alternating low energy and elevated energy acquisition mode. The continuum spectra acquisition time in each mode was 1.5 s with a 0.1 s interscan delay. In the low-energy MS mode, data were collected at constant collision energy of 3 eV. In the elevated-energy MS mode, the collision energy was increased from 12 to 45 eV during each 1.5 s spectrum. The radiofrequency that was applied to the quadrupole mass analyzer was adjusted such that the ions from 50 to 2000 m/z were efficiently transmitted, which ensured that any ions less than 50 m/z observed in the LC-MS data were only derived from dissociations in the TRAP T-wave collision cell.

### Data processing and protein identification

The MS data that were obtained from the LC-MS^E^ analysis were processed and searched using the ProteinLynx Global Server (PLGS) version 2.5 (Waters, Manchester, UK). Proteins were identified using the software’s embedded ion accounting algorithm and a search of the Glycine max database with MassPREP digestion standards (MPDS) UniProtKB/Swiss-Prot sequences (Phosphorylase - P00489 - PHS2_RABIT, Bovine Hemoglobin - P02070 - HBB_BOVIN, ADH - P00330 - ADH1_YEAST, BSA - P02769 - ALBU_BOVIN) that were appended to the database. Identifications and quantitative data packaging were performed using dedicated algorithms [28,31] and a search against a soybean Uniprot database. The ion detection, clustering, and log-scale parametric normalizations were performed in PLGS with an ExpressionE license installed. The intensity measurements were typically adjusted for these components, i.e., the deisotoped and charge state-reduced EMRTs that were replicated throughout the entire experiment for the analysis at the EMRT cluster level. The fixed modification of carbamidomethyl-C was specified, and the included variable modifications were acetylation of the N-terminus, deamidation of N, deamidation of Q and oxidation of M. Components were typically clustered with a 10ppm mass precision and a 0.25 min time tolerance against the database-generated theoretical peptide ion masses with a minimum of one matched peptide. The alignment of elevated-energy ions with low-energy precursor peptide ions was performed with an approximate precision of 0.05 min. One missed cleavage site was allowed. The precursor and fragment ion tolerances were determined automatically. The protein identification criteria also included the detection of at least three fragment ions per peptide, 6 fragments per protein and the determination of at least one peptide per protein; the identification of the protein was allowed with a maximum 4% false positive discovery rate in at least four technical replicate injections. Using protein identification replication as a filter, the false positive rate was minimized because false positive protein identifications, i.e., chemical noise, have a random nature and do not tend to replicate across injections. For the analysis of the protein identification and quantification level, the observed intensity measurements were normalized to the intensity measurement of the identified peptides of the digested internal standard. Protein tables generated by PLGS were merged, and the dynamic range of the experiment was calculated using the in-house software program MassPivot by setting the minimum repeat rate for each protein in all replicates to 2.

### Uniprot soybean database digestion and experiment analysis

*Glycine max* protein sequences were obtained from Uniprot ( http://www.uniprot.org), and the theoretical tryptic digestion was performed using the in-house software Digestion tool. The digestion was performed allowing 1 missed cleavage, and the molecular mass and isoelectric point of all peptides and proteins were calculated. The peptide and protein tables from PLGS were compared with the database digestion table using the Spotfire software ( http://spotfire.tibco.com/), suitable graphics were generated for all data. Microsoft Excel (Microsoft, USA) was used for table manipulations.

## Authors’ contributions

All authors contributed equally to this work. All authors read and approved the final manuscript.
